# The Value of Ortho-ID Teams in Treating Bone and Joint Infections

**DOI:** 10.7150/jbji.41663

**Published:** 2019-11-20

**Authors:** Shawn Vasoo, Monica Chan, Parham Sendi, Elie Berbari

**Affiliations:** 1 National Centre for Infectious Diseases, Singapore; 2 Department of Infectious Diseases, Tan Tock Seng Hospital, Singapore; 3 Lee Kong Chian School of Medicine, Nanyang Technological University, Singapore; 4 Department of Infectious Diseases and Hospital Epidemiology, University Hospital Basel, University of Basel; 5 Department of Orthopaedics and Traumatology, University Hospital Basel, University of Basel; 6 Institute of Infectious Diseases, University of Bern, Bern, Switzerland; 7 Division of Infectious Diseases, Mayo Clinic, Rochester, MN, USA; 8 Mayo Clinic College of Medicine, Rochester, MN, USA

## Introduction

In this issue of *JBJI*, Sven Åke Hedström and Lars Lidgren, two founding members of the European Bone and Joint Infection Society (EBJIS), report about their cooperation between orthopaedic surgeons and infection specialists in bone and joint infections in 1982 1. Due to their pioneering work, the value of Infectious Diseases (ID) specialists has found is essential ground in Bone and Joint Infections (BJIs).

BJIs are associated with significant morbidity and are a growing global burden. Besides classic ID such as spinal tuberculosis which have afflicted humans for centuries, and still do in the 21^st^ century, newer “maladies” such as orthopaedic implant associated infections (OIAIs) are set to rise with improved life expectancy and the corresponding number of prosthetic joint surgeries performed 2. In this editorial, we aim to review the development of the expertise in the field of bone and joint infections from various perspectives.

## Historical development of BJI units

Expertise in modern BJI units grew with advances made in the disciplines of orthopaedic surgery, infectious diseases, clinical microbiology and antibiotic pharmacotherapy 3-5, alongside infection prevention and control, pathology, plastic surgery, vascular surgery, anaesthesiology and radiology. The advent of sulfonamides and penicillin in the 1940s dramatically improved the treatment of osteomyelitis, which till then was predominantly treated surgically with drainage and rest (or the “Orr Method” as described by Dr. H. Winnett Orr)^3^,^4^ with less conventional therapies such as maggot therapy, bacteriophages, refrigeration and salt-water pool therapy as adjuncts. The first prosthetic knee joint was implanted by the German surgeon Themistocles Gluck in 1890 for a joint ravaged by tuberculosis, with subsequent pioneering work in knee and hip arthroplasties by Charney and Insall in the 1960s and 1970s, while fracture-fixation with wires was described as early as 1775 6. While these were a boon to restore functionality, it became apparent quickly that infections associated with these prostheses could be devastating, spurring research in preventive strategies (such as antisepsis), diagnosis and treatment, with much of the early work driven by surgeons such as Lister^6^, and orthopaedic surgeons such as Charney 6.

Given the multi-disciplinary approach needed to prevent, diagnose and treat BJIs, and expertise needed to optimise outcomes, units and bed wards dedicated to the treatment of BJIs have developed in facilities which are referral centers and who typically perform a high volume of surgeries related to BJIs. A non-exhaustive list of examples of such units include the Bone Infection Unit, at the Nuffield Orthopaedic Centre, Oxford, UK, the Wrightington Hospital Orthopaedic Centre of Excellence, Lancashire, UK, the Basel University Medical Clinic and Interdisciplinary Unit for Orthopaedic Infections, Liestal, Switzerland, and the Orthopaedic Infectious Diseases Section at the Mayo Clinic, Rochester, MN. In the UK for example, it has been proposed that ~3-6 networks be formed nationally, with each served by a multidisciplinary specialist BJI unit comprising of orthopaedists and ID physicians specializing in BJI for patients requiring complex revisions (e.g. Ilizarov techniques) or who have multidrug resistant infections and multiple medical comorbidities. Such units are also supported by plastic surgeons with experience in complex microsurgical reconstruction, vascular surgeons who optimise perfusion and healing, microbiology laboratory support and outpatient antimicrobial therapy services 7. In addition, many tertiary medical centres world-wide have recognised the importance of close liaison between orthopaedists and infectious disease specialists with formal training or an interest in BJIs and other complex musculoskeletal infections.

## Frequency and epidemiology of bone and joint infection in clinical practice

While prosthetic joint infections (PJIs) are relatively uncommon in proportion to the volume of surgeries performed (~1-2% overall)^8^, and BJIs as a proportion of all hospitalizations (0.2% nationally in one French study 9), consultations for musculoskeletal infections, in general, are very common in a hospital ID practice. The same French study found that BJI accounted for significant morbidity and impact clinically and economically - with a prevalence of 54 per 100,000 nationally 9. Studies in different countries and time periods have attempted to capture the volume of ID consultations for musculoskeletal infections in various ways - these have accounted from 7% up to 30% of all ID consultations 10-12 and a consultation rate of 7.6 per 100 admissions arising from the orthopaedics department in one Israeli study 10. BJIs are often the most common diagnosis for OPAT, as described in a Singaporean study (osteomyelitis being the most common indication, comprising 15% of the caseload) 13 and Dutch study (BJI comprised 38%) 14, with another UK study finding that 15% of emergency admissions and referrals (excluding PJI) were associated with BJIs or soft tissue infection 15.

## Impact and value of infectious diseases consultation on BJIs

**Figure 1:**
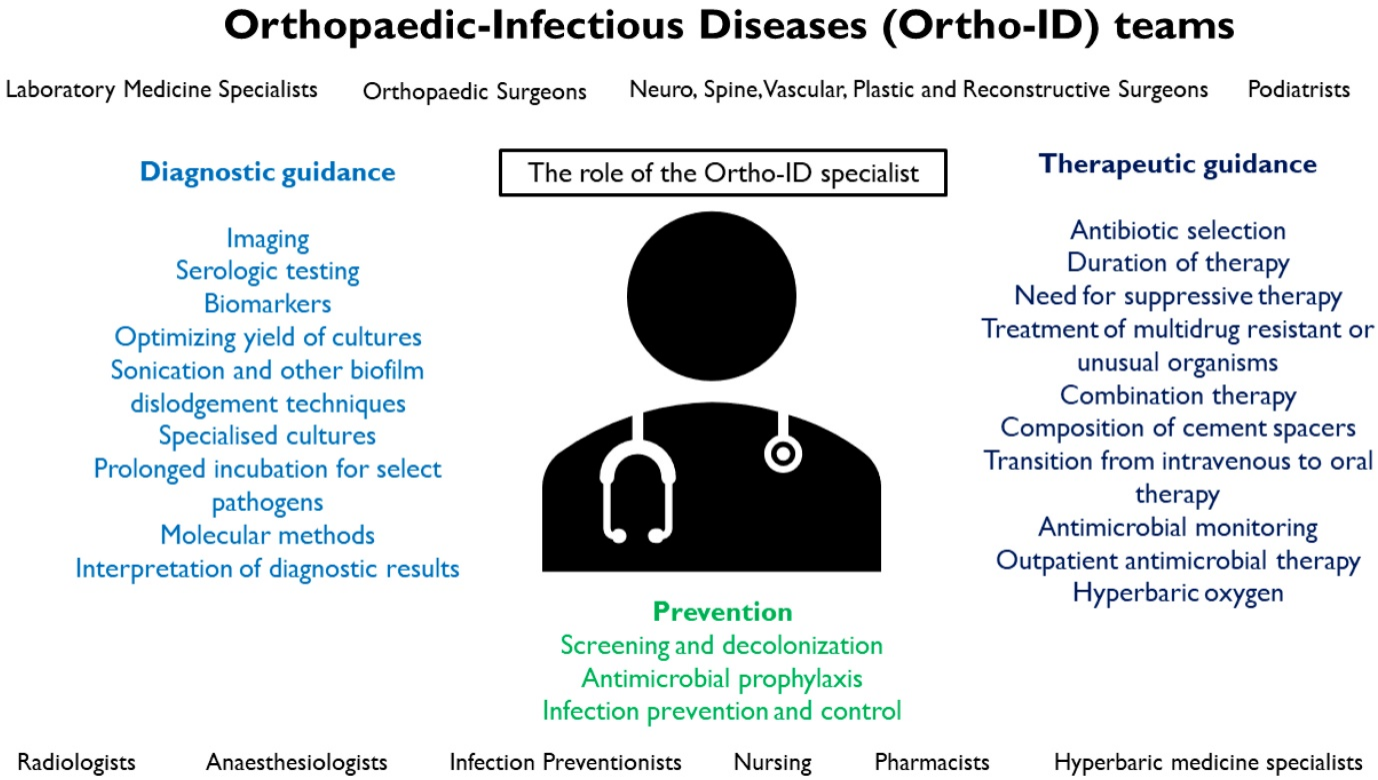
The Role of the Ortho-ID specialist

The ID physician with expertise in BJIs may bring the following benefits to a patient's management (Figure 1):

- Recommendation of suitable diagnostic testing and adjunctive investigations e.g. sonication, prolonged incubation, molecular methods, biomarkers, relevant imaging, serologic testing depending on epidemiological risk factors and presentation, and expertise in interpretation of these results
- Expertise in guiding therapy, e.g. selection of appropriate antibiotic and duration based on available microbiological results and preferred antibiotic choices, treatment of complicated BJIs including those caused by MDROs or unusual organisms, combination therapy with rifampicin-containing regimens for staphylococcal PJIs and osteomyelitis, peri-operative antibiotic prophylaxis, composition of antibiotic cement spacers used intraoperatively, transitions from IV to oral therapy, outpatient antimicrobial therapy, other adjuncts such as hyperbaric oxygen therapy.
- Monitoring and follow-up, e.g. ensuring tolerability of treatment, minimising side effects, identifying complications and providing alternatives where necessary, determining duration including long-term antimicrobial suppressive therapy

Improvement in systems processes in the prevention, diagnosis and treatment of BJIs in conjunction with the orthopaedist, vascular surgeons, clinical microbiologists, anaesthesiologists and infection preventionists. Examples of initiatives include prevention of BJIs via screening and decolonization for *S. aureus* prior to surgery, optimizing and in select cases, individualizing antibiotic prophylactic regimens for surgery, improving diagnostic yield for BJI by standardizing optimizing sampling (e.g. adequate number and type of intraoperative samples, culture methodology), antibiotic stewardship by determining necessity for continued antibiotics or transitioning from IV to oral antibiotics.

Multiple studies have demonstrated a benefit of ID consultations for MRSA bacteremia with reported reductions in mortality and relapse 16. Similar mortality benefits have been noted for ID consultation in multi-drug resistant organism infections in one study which included BJIs 16. Few studies have described the impact of infectious disease consultation for BJIs specifically; however, one 2015 study found that osteomyelitis cases managed by a orthopaedist alone (without an ID physician) were 4.6 times more likely to relapse 16. A more recent analysis of administrative claims data of over 70,000 index stays for a broad range of common ID conditions found that early ID intervention was associated with shorter hospital stays, lower readmission rates and overall costs^17^. Specifically for BJIs, during initial hospitalization, patients with osteomyelitis and septic arthritis had lower costs in the initial hospitalization if they had an early ID consult (versus no or late ID consult).

**Education of the next generation of Orthopaedic infectious diseases providers**Most infectious diseases training programmes provide training in Orthopaedic infectious diseases as part of fellowship training. There are however few formal/dedicated training programmes. In the US, one such non-ACGME (Accreditation Council for Graduate Medical Education) programme is available at the Mayo Clinic in Rochester, MN, USA (1-year subspecialty orthopaedic infectious diseases fellowship). Emphases of the fellowship include participation in multidisciplinary team management of patients with both musculoskeletal infections from the common to complex and research opportunities in BJIs. Other high-volume BJI units regularly receive physician-trainees or fellows for further clinical training and research, and these are important in the building up of expertise and future generations of orthopaedic infectious diseases providers. The EBJIS has recognized this opportunity and organizes travelling fellowships since 2014^18^. Each year, three fellows among all applicants will be awarded and offered the chance to visit three or four distinguished European Centers specializing in BJIs.

## Societies that focus on BJIs

At the societal level, professional bodies dedicated bodies to advancing knowledge and best practices in the management of musculoskeletal infections besides the EBJIS (founded 1993) include the Musculoskeletal Infection Society (MSIS, founded 1989) and International Working Group on the Diabetic Foot (IWGDF, founded 1996). These, alongside other speciality professional societies (for e.g. the Infectious Diseases Society of America (IDSA), the European Society of Clinical Microbiology and Infectious Diseases (ESCMID), the European Society of Paediatric Infectious Diseases (ESPID), European Association of Nuclear Medicine (EASN), the American Academy of Orthopaedic Surgeons (AAOS) and consensus groups (e.g. the International Consensus Meeting (ICM) on surgical‐site infection and periprosthetic joint infection) have promoted dialogue, research, consensus and promotion of evidenced-based practise through various guidelines and meetings, covering important areas such as diagnostic tools and criteria for PJIs and its management, and other BJIs 19-23. Several BJI-specific scientific meetings are also held regularly - these include the Oxford Bone Infection Conference (OBIC), MSIS and EBJIS meetings.

## Definition and Classifications - the basis for translational and clinical studies

For many decades, providers classified and categorized BJIs. The heterogeneity of cases and definitions in clinical practice and attendant clinical data caused a burden for useful conclusions. The collaborations of orthopaedic and trauma surgeons and ID physicians have led to a continuously evolving and improved structure in defining and understanding osteomyelitis of long bones 24,25, fracture related infections 26 and prosthetic joint infections^22^ .

## Gaps and future directions

The recent decades have seen strides in the management of BJIs. From a scientific and clinical perspective, these fall into distinct areas as nicely outlined by the ICM: microbiology/antimicrobials, biomaterials/implants, surgery/clinical care, and immunology/host immunity (such as vaccines to prevent BJIs), although the last has been the most nascent in terms of scientific discovery/breakthroughs, and should receive more attention and funding 19. From a clinical front, recent trials such as OVIVA which have questioned traditional paradigms such as the length of intravenous antimicrobial therapy for osteomyelitis are potential game-changers and a reminder that specialists in BJI are best-placed, as a community, to conduct these pivotal trials and gather evidence which will inform practice 27. With evolving technology, the field would need to also address the place of newer techniques such as metagenomics and proteomics, and novel biomarkers for the diagnosis of BJIs. Antimicrobial resistance and a lack of suitable antimicrobial therapy, particularly in chronic and biofilm-associated infections, is also an area which the orthopaedic infectious diseases specialists need to confront. Most importantly, greater efforts need to be put into preventive strategies, which are always 'better than cure'. Some of these would entail new scientific discoveries; however a lot of these may be done with what we already know - such as waging the war on diabetes (and its attendant global burden of foot infections).

Orthopaedic ID has matured into a subspecialty in its own right. National and international societies should continue to develop formal training programs, certification, and recognise centers with excellence in this field. The management of BJIs is often nuanced and complex and the Orthopaedic ID subspecialists bring much to the table in the prevention and management of BJIs.
